# Supplementary material for: A Zipf-plot based normalization method for high-throughput RNA-seq data
Source: PLoS One. 2020 Apr 9;15(4):e0230594. doi: 10.1371/journal.pone.0230594 (PMC7144957; doi:10.1371/journal.pone.0230594)
Supplement: Attachment — Submitted filename: R2R.docx [file pone.0230594.s001.docx]

**In the following rebuttal letter, the point-to-point responses are highlighted in bold fonts.**

You are being carbon copied ("cc:'d") on an e-mail "To" "Bin Wang" [bwang@southalabama.edu](mailto:bwang@southalabama.edu)

CC: [bin.wang@fda.hhs.gov](mailto:bin.wang@fda.hhs.gov), [bxw28@case.edu](mailto:bxw28@case.edu)

PONE-D-19-32855

A normalization method for next generation sequencing data using power transformation

PLOS ONE

Dear Dr. Wang,

Thank you for submitting your manuscript to PLOS ONE. After careful consideration, we feel that it has merit but does not fully meet PLOS ONE’s publication criteria as it currently stands. Therefore, we invite you to submit a revised version of the manuscript that addresses the points raised during the review process.

==============================

The author needs to study/review more on current popular normalization approaches and tools for RNA-seq data. More extensive comparisons are needed in the manuscript to show the performance of proposed method.

**Five recent publications have been reviewed and the contents have been updated, especially in the Introduction and Discussion sections. The proposed method has also been benchmarked with four popular linear scaling normalization methods including Total Count (TC), Upper Quartile (UQ), Trimmed Mean of M-values (TMM), Relative Log Expression (RLE), and one nonlinear normalization method – the Quantile Normalization (QN) method.**

==============================

We would appreciate receiving your revised manuscript by Feb 21 2020 11:59PM. When you are ready to submit your revision, log on to <https://www.editorialmanager.com/pone/> and select the 'Submissions Needing Revision' folder to locate your manuscript file.

If you would like to make changes to your financial disclosure, please include your updated statement in your cover letter.

To enhance the reproducibility of your results, we recommend that if applicable you deposit your laboratory protocols in [protocols.io](http://protocols.io/), where a protocol can be assigned its own identifier (DOI) such that it can be cited independently in the future. For instructions see: <http://journals.plos.org/plosone/s/submission-guidelines#loc-laboratory-protocols>

Please include the following items when submitting your revised manuscript:

- A rebuttal letter that responds to each point raised by the academic editor and reviewer(s). This letter should be uploaded as separate file and labeled 'Response to Reviewers'.
- A marked-up copy of your manuscript that highlights changes made to the original version. This file should be uploaded as separate file and labeled 'Revised Manuscript with Track Changes'.
- An unmarked version of your revised paper without tracked changes. This file should be uploaded as separate file and labeled 'Manuscript'.

Please note while forming your response, if your article is accepted, you may have the opportunity to make the peer review history publicly available. The record will include editor decision letters (with reviews) and your responses to reviewer comments. If eligible, we will contact you to opt in or out.

We look forward to receiving your revised manuscript.

Kind regards,

Xiaofeng Wang

Academic Editor

PLOS ONE

Journal Requirements:

When submitting your revision, we need you to address these additional requirements.

1. Please ensure that your manuscript meets PLOS ONE's style requirements, including those for file naming. The PLOS ONE style templates can be found at

<http://www.journals.plos.org/plosone/s/file?id=wjVg/PLOSOne_formatting_sample_main_body.pdf> and <http://www.journals.plos.org/plosone/s/file?id=ba62/PLOSOne_formatting_sample_title_authors_affiliations.pdf>

2. Thank you for stating the following in the Acknowledgments Section of your manuscript:

"This work was supported by a grant from the Simons Foundation (#422535, Bin Wang)."

We note that you have provided funding information that is not currently declared in your Funding Statement. However, funding information should not appear in the Acknowledgments section or other areas of your manuscript. We will only publish funding information present in the Funding Statement section of the online submission form.

Please remove any funding-related text from the manuscript and let us know how you would like to update your Funding Statement. Currently, your Funding Statement reads as follows:

"This research was funded and terminated due job change for the corresponding author. Grant number provided in the manuscript."

**The funding information has been removed from the Acknowledgement Section and I wish to update the Funding Statement as follows: “This work was supported by a grant from the Simons Foundation (#422535, Bin Wang).”**

**Thanks.**

[Note: HTML markup is below. Please do not edit.]

Reviewers' comments:

Reviewer's Responses to Questions

**Comments to the Author**  1. Is the manuscript technically sound, and do the data support the conclusions?  The manuscript must describe a technically sound piece of scientific research with data that supports the conclusions. Experiments must have been conducted rigorously, with appropriate controls, replication, and sample sizes. The conclusions must be drawn appropriately based on the data presented.

Reviewer #1: Yes

Reviewer #2: Yes

2. Has the statistical analysis been performed appropriately and rigorously?

Reviewer #1: Yes

Reviewer #2: Yes

3. Have the authors made all data underlying the findings in their manuscript fully available?  The [PLOS Data policy](http://www.plosone.org/static/policies.action#sharing) requires authors to make all data underlying the findings described in their manuscript fully available without restriction, with rare exception (please refer to the Data Availability Statement in the manuscript PDF file). The data should be provided as part of the manuscript or its supporting information, or deposited to a public repository. For example, in addition to summary statistics, the data points behind means, medians and variance measures should be available. If there are restrictions on publicly sharing data—e.g. participant privacy or use of data from a third party—those must be specified.

Reviewer #1: Yes

Reviewer #2: Yes

4. Is the manuscript presented in an intelligible fashion and written in standard English?  PLOS ONE does not copyedit accepted manuscripts, so the language in submitted articles must be clear, correct, and unambiguous. Any typographical or grammatical errors should be corrected at revision, so please note any specific errors here.

Reviewer #1: Yes

Reviewer #2: Yes

5. Review Comments to the Author  Please use the space provided to explain your answers to the questions above. You may also include additional comments for the author, including concerns about dual publication, research ethics, or publication ethics. (Please upload your review as an attachment if it exceeds 20,000 characters)

Reviewer #1: This manuscript focuses on the proposal of a normalization method for RNA-seq data analysis. It is well written and reasonably sophisticated. Yet I have a couple of comments regarding its publication in PLOS ONE.

Major comment:

1. The first paragraph in Introduction Section mentions measurement errors. Maybe the author can add one or few sentences to explain what are the sources of measurement errors in next generation sequencing data? This added explanation may also help in making a connection between the first paragraph and the second paragraph.

**Thanks for point this out. More information has been added about the errors in NGS data including the sequencing errors and measurement errors.**

2. Since this proposed normalization method assumes that all gene profile distributions have similar upper tail behaviors, can the author provide the frequency distribution in the upper tail area of the RNA-seq data for LCL to see if this assumption holds?

**Thanks for the recommendation. To show the similarity among the different profiles, we added two plots to Figure 1 showing the empirical distribution functions on the raw data and log-transformed scale in plot (a) and (b), respectively. In addition, we produced a plot showing the relationship between the Zipf plot in plot (c) and a modified Zipf plot, in plot (d) using log(ECDF(x)) instead of log(Rank). Comments have been added to specify the similarity of upper tails behaviors of all profiles in Figure 1. The new figures have been explained in detail.**

3. Math symbol convention: suggest using different math symbols/notations for different mathematical formulas:

Equation (1): uses j = 1 to n and uses j = 1 to i

**Thanks. “j=1 to n” was used to ensure all gene expressions will be taken into consideration. “j=1 to i” was used because fj is the frequency of zj which had been sorted.**

Figure 1: x-axis label says Log(X), it should be Log(zi), right?

**The Zipf plot shows the relationship between Log(Rank(X)) and Log(X). The plot was drawn using Log(ri) against Log(xi). We changed the labels for the x-axis and y-axis to Log(zi) and Log(ri). Thanks.**

Line 91: suggest using a symbol other than x since x has been used in previous formulas.

**Thanks for raising this concern. We added a comment about the reuse of the notation to avoid possible confusion.**

4. Equation (2): it seems that the density function shown here is incorrect, assuming that the cumulative distribution function is correct. Please check.

**You are right. There is a type error in the pdf. The pdf has been corrected. We double-checked and verified that this change won’t cause changes to the other places. Thanks.**

5. Equation (3): it should be an approximate relationship, not an exact relationship (not exactly equal), right?

**Correct. The equal sign has been changed to approximately equal sign. Thanks.**

6. I am curious about the performance of the proposed method on gene profiles other than profile 47 and profile 107. Has the author done the data analysis?

**More results have been added by applying the proposed method on all profiles and compared the results with some established population normalization methods. Thanks.**

7. Could the author provide the rationale that why the optimal estimates should be the pair that minimize the maximum distance between the two empirical distributions?

**Thanks for pointing this out. The main idea behind that is to make the two distributions as close as possible after normalization. The Kolmogorov-Smirnov statistics is used to measure the distance between the two CDFs due to the simplicity of its form and ease to compute. Comments have been added to justify the rationale of choosing the estimates that minimize the maximum distance between the two empirical distributions.**

Minor comment:

1. The terminology ‘global profile’ was used in the abstract but not introduced in the main manuscript. Maybe the author can explain what does ‘global profile’ mean?

**Thanks. We meant the global information of the profile. This has been corrected.**

2. Line 21: what do loess and MA-plot stand for?

**A brief introduction has been added about the MA-plot.**

3. Line 73: m distinct values are the actual distinct values or all possible distinct values? If it is all possible distinct values, then the distinct value number m is the same across all n genes. If it is actual distinct values, the distinct value number m can vary from one gene to another, right?

**The value of m represents the actual distinct values in a profile. The value of m is usually much smaller than n, which the total number of genes. It is true that the number of distinct value varies from sample to sample. It is also true that the distinct values of genes vary gene by gene if we the expression profiles for different genes. Therefore, we use m_x and m_y in for the two profiles to be normalized in lines 91 and 93.**

4. The curves in Figure 2 are hard to see. Maybe the author can use other colors for the dotted lines and the dashed lines?

**Thanks. We reproduced Figure 2 by changing the line type from dotted to dash-dotted. The line width were increased as well.**

5. It is great that all the algorithms have been implemented in R. Yet I tried and found that the package bda is not available for download purpose. Maybe the author can provide further clarification for the R code implementation in the Conclusion Section.

**The R package bda has been rebuilt by removing the Fortran subroutines. The new version has been approved and the new package has been submitted to CRAN and will become available soon. Comments have been added about the R function to implement the proposed normalization method. Sample codes can be found in bda.**

Minor concern:

1. The second paragraph in Introduction Section lacks intention?

**Changes have been made to the second paragraph to include more details about the existing normalization methods and commented on their pros and cons.**

2. In Abstract: ‘the proposed normalization method use global profile information’ -> ‘the proposed normalization method uses global profile information’.

**Changed. The whole manuscript has been carefully proofread.**

Reviewer #2: The author developed a Zipf plot based normalization method for RNA-seq data analysis by assuming similar upper tail distributions in all gene profiles. It takes the zero-inflated feature of RNA-seq data into account, and the results indicate that the proposed method improves data distributions. However, I have some major concerns:

1) The references in the manuscript are not state of the art. A lot of newly developed normalization methods for RNA-seq data in the past decade are not mentioned/discussed. Here are some papers: [1] Dillies. MA, et al. A comprehensive evaluation of normalization methods for Illumina high-throughput RNA sequencing data analysis. Briefings in bioinformatics, 2013. [2] Li, P, et al. Comparing the normalization methods for the differential analysis of Illumina high-throughput RNA-Seq data. BMC bioinformatics, 2015. [3] Zyprych-Walczak, J., et al. The impact of normalization methods on RNA-Seq data analysis. BioMed research international, 2015. [4] Abbas-Aghababazadeh, Farnoosh, et al. Comparison of normalization approaches for gene expression studies completed with high-throughput sequencing. PloS one, 2018.

**Thank you for the information. We search the literature and significantly updated the introduction section to provide the latest development in related fields. The proposed normalization method focuses on linear recalling normalization so we didn’t discuss the data-driven normalization and differentially-expressed gene detection methods. In the revised version, we added new results about the performances of the existing normalization methods in various applications.**

2) The author should compare with those existing normalization methods (especially the one in DESeq2, which is the most popular tool for RNA-seq data analysis at the current stage) extensively in the manuscript to show the performance of the proposed method.

**Thanks for the suggestion. In the revised version, we benchmarked the performances of the proposed method with multiple existing popular methods. The performances were further discussed in the Discussion Section.**

Minor comments:

The English writing is overall good, but there are still some grammatical mistakes. Please carefully proofread.

**Thanks for point this out. We have carefully proofread the revised version.**
