# Supplementary material for: A Zipf-plot based normalization method for high-throughput RNA-seq data
Source: PLoS One. 2020 Apr 9;15(4):e0230594. doi: 10.1371/journal.pone.0230594 (PMC7144957; doi:10.1371/journal.pone.0230594)
Supplement: Attachment — Submitted filename: Response.docx [file pone.0230594.s002.docx]

We provide the point-to-point response (**in blue**) to the reviewers’ comments as follows.

6. Review Comments to the Author

Please use the space provided to explain your answers to the questions above. You may also include additional comments for the author, including concerns about dual publication, research ethics, or publication ethics. (Please upload your review as an attachment if it exceeds 20,000 characters)

Reviewer #1: I appreciate the author for carefully addressing the reviewers' comments and significantly revising the manuscript. Yet I still have two comments regarding the presentation flow/the writing.

1. Thank the author for rewriting the introduction section to include the existing normalization methods for RNA-seq data. However I think these existing methods are not clearly described in the introduction section in terms of (a) the underlying assumptions (b) applicable scenarios (c) pros and cons (d) implementation and it is difficult to read paragraph 2, paragraph 3 and paragraph 4.

**We really appreciate your patience and valuable suggestions on further improving the manuscript. Paragraphs 2—4 of the Introduction section have been reorganized and rewritten to improve readability. The changes are highlighted in the revised manuscript with track changes.**

2. I agree that this manuscript needs further modifications in the presentation flow and in the language, especially in those newly added parts. For example, the author uses the phrases like 'we can see', 'we see', 'we want' a lot yet it can be better expressed. Also some phrases are not very formal, for example, Line 173 – Line 174 says that 'and manipulate the Zipf plot of profile Y' but the word 'manipulate' could perhaps be replaced by an alternative word suitable for this setting.

**Thanks for pointing this out. Global changes have been made accordingly.**

Reviewer #2: The author has carefully and comprehensively revised the manuscript in response to my comments. I do think that the paper has been improved considerably. I do not have any major comments on the revised manuscript, but a few minor concerns:
1. I appreciate the author's extensive discussion and comparison with other RNA-seq normalization methods. I think the message of how to select appropriate methods in the Conclusion section is important. I would suggest adding a brief sentence of this conclusion to the end of Abstract.

**Some sentences in the Abstract have been rewritten and more information has been added about the two normalization schemes of the proposed normalization method. Thank you.**

2. At the end of Introduction, the author says "This new normalization method helps to prepare high-quality data for further normalization or down-streaming analyses such as classification or clustering analysis." But no data of a further normalization, classification or clustering are shown. The author may reconsider this statement.

**We agree with your comments. This sentence has been removed. Thanks.**
